# Supplementary material for: Human microbiome variation associated with race and ethnicity emerges as early as 3 months of age
Source: PLoS Biol. 2023 Aug 17;21(8):e3002230. doi: 10.1371/journal.pbio.3002230 (PMC10434942; doi:10.1371/journal.pbio.3002230)
Supplement: S1 Text — (DOCX) [file pbio.3002230.s001.docx]

**Supplementary text**

*Impact of age on gut microbiome composition and diversity*

Differences between within-sample (alpha) diversity were assessed using five metrics – Faith’s phylogenetic diversity (PD), observed amplicon sequence variants (ASVs), Chao 1, Shannon diversity, and Pielou’s evenness indices. As expected, age had a significant effect on all alpha diversity indices when controlling for study and individual identity (LME: all p<0.001) (Figs 1A and S22 and S4 Table). Across all age categories, race had a significant effect on three of the five within-sample alpha diversity indices tested (LME: Faith’s PD: χ^2^=5.000, p=0.082; Observed ASVs: χ^2^=12.579, p=0.002; Chao 1: χ^2^=15.531, p<0.001; Shannon: χ^2^=9.609, p=0.008; Pielou’s: χ^2^=4.297, p=0.117), and ethnicity had a significant effect for Faith’s PD (LME: Faith’s PD: χ^2^=4.651, p=0.031; Observed ASVs: χ^2^=2.839, p=0.092; Chao 1: χ^2^=2.781, p=0.095; Shannon: χ^2^=1.151, p=0.283; Pielou’s: χ^2^=0.010, p=0.922) (S23-24 Figs and S4 Table). Pairwise comparisons showed that Black individuals had significantly higher alpha diversity compared with White individuals across all age categories (S4 Table). Asian/Pacific Islander individuals did not differ significantly from other groups likely because of small sample sizes for this group.

When accounting for the effects of study and individual differences and including all variables of interest (race, ethnicity, age, sex, delivery mode, and infant diet) in the models, age has the largest association with gut microbiome composition using both unweighted UniFrac distances (PERMANOVA: F_5,2286_=42.473, R^2^=0.052, p=0.002) and weighted UniFrac distances (PERMANOVA: F_5,2286_=64.080, R^2^=0.079, p=0.002) (Figs 1 and S1 and S2 Table), as expected (1–6). When examining all age categories together, race and ethnicity have marginal but significant associations with gut microbiome composition using both unweighted UniFrac distances (PERMANOVA: p=0.070) and weighted UniFrac distances (PERMANOVA: p=0.004) (S2 Fig and S2 Table). Ethnicity has a significant association with gut microbiome composition using unweighted UniFrac distances only (PERMANOVA: p=0.024) (S2 Fig and S2 Table). All pairwise comparisons were significant for race (p<0.05) (S3 Table).

*Impact of additional factors on gut microbiome composition and diversity*

Delivery mode (Cesarian section, vaginal) and infant diet (exclusively breastfed, exclusively formula fed, mixed formula and breastfed) were significantly associated with gut microbiome composition when controlling for the effects of study differences and including only one sample per individual using unweighted UniFrac distances (PERMANOVA: Delivery: F_1,428_=1.345, R^2^=0.003, p=0.024; Diet: F_1,428_=2.481, R^2^=0.011, p=0.026) (S3-S9 Figs and S2 Table). Sex and infant diet were also associated with differences in Shannon diversity and observed ASVs (S25-S26 Figs and S4 Table).

Multiple studies of the childhood microbiome have found associations between infant feeding history and gut microbiome composition and diversity (4,5,7–14), though whether gut microbial diversity increases or decreases when infants are exclusively breastfed is unclear (9,13). We did not find a strong association between gut microbiome composition and birth mode in our analysis. While many studies have indicated that infants born via caesarian delivery have altered gut microbiomes (8,10–12,15–18), others find no relationship between delivery mode and gut microbiome composition (4,7,13,14,19) and one found that the association between delivery mode and the gut microbiome did not persist past 14 months of age (5).

**References**

1. Hollister EB, Riehle K, Luna RA, Weidler EM, Rubio-Gonzales M, Mistretta TA, et al. Structure and function of the healthy pre-adolescent pediatric gut microbiome. Microbiome. 2015 Aug 26;3(1):36.

2. Koenig JE, Spor A, Scalfone N, Fricker AD, Stombaugh J, Knight R, et al. Succession of microbial consortia in the developing infant gut microbiome. Proceedings of the National Academy of Sciences. 2011;108 Suppl 1:4578–85.

3. Lim ES, Zhou Y, Zhao G, Bauer IK, Droit L, Ndao IM, et al. Early life dynamics of the human gut virome and bacterial microbiome in infants. Nature Medicine. 2015 Oct;21(10):1228–34.

4. Planer JD, Peng Y, Kau AL, Blanton LV, Ndao IM, Tarr PI, et al. Development of the gut microbiota and mucosal IgA responses in twins and gnotobiotic mice. Nature. 2016 Jun;534(7606):263–6.

5. Stewart CJ, Ajami NJ, O’Brien JL, Hutchinson DS, Smith DP, Wong MC, et al. Temporal development of the gut microbiome in early childhood from the TEDDY study. Nature. 2018;562(7728):583–8.

6. Chen DW, Garud NR. Rapid evolution and strain turnover in the infant gut microbiome. Genome Res. 2022 Jun 1;32(6):1124–36.

7. Cioffi CC, Tavalire HF, Neiderhiser JM, Bohannan B, Leve LD. History of breastfeeding but not mode of delivery shapes the gut microbiome in childhood. PLOS ONE. 2020 Jul 2;15(7):e0235223.

8. Coker MO, Laue HE, Hoen AG, Hilliard M, Dade E, Li Z, et al. Infant Feeding Alters the Longitudinal Impact of Birth Mode on the Development of the Gut Microbiota in the First Year of Life. Front Microbiol. 2021;12:642197.

9. Gschwendtner S, Kang H, Thiering E, Kublik S, Fösel B, Schulz H, et al. Early life determinants induce sustainable changes in the gut microbiome of six-year-old children. Scientific Reports. 2019;9:12675.

10. Levin AM, Sitarik AR, Havstad SL, Fujimura KE, Wegienka G, Cassidy-Bushrow AE, et al. Joint effects of pregnancy, sociocultural, and environmental factors on early life gut microbiome structure and diversity. Scientific Reports. 2016 Aug 25;6(1):31775.

11. Penders J, Thijs C, Vink C, Stelma FF, Snijders B, Kummeling I, et al. Factors Influencing the Composition of the Intestinal Microbiota in Early Infancy. Pediatrics. 2006 Aug 1;118(2):511–21.

12. Sordillo JE, Zhou Y, McGeachie MJ, Ziniti J, Lange N, Laranjo N, et al. Factors influencing the infant gut microbiome at age 3-6 months: Findings from the ethnically diverse Vitamin D Antenatal Asthma Reduction Trial (VDAART). Journal of Allergy and Clinical Immunology. 2017 Feb 1;139(2):482-491.e14.

13. Stearns JC, Zulyniak MA, de Souza RJ, Campbell NC, Fontes M, Shaikh M, et al. Ethnic and diet-related differences in the healthy infant microbiome. Genome Med. 2017 Mar 29;9(1):32.

14. Zhong H, Penders J, Shi Z, Ren H, Cai K, Fang C, et al. Impact of early events and lifestyle on the gut microbiota and metabolic phenotypes in young school-age children. Microbiome. 2019;7(1):2.

15. Busi SB, de Nies L, Habier J, Wampach L, Fritz JV, Heintz-Buschart A, et al. Persistence of birth mode-dependent effects on gut microbiome composition, immune system stimulation and antimicrobial resistance during the first year of life. ISME Communications. 2021 Mar 26;1:8.

16. Dominguez-Bello MG, Costello EK, Contreras M, Magris M, Hidalgo G, Fierer N, et al. Delivery mode shapes the acquisition and structure of the initial microbiota across multiple body habitats in newborns. Proceedings of the National Academy of Sciences. 2010;107(26):11971–5.

17. Lundgren SN, Madan JC, Emond JA, Morrison HG, Christensen BC, Karagas MR, et al. Maternal diet during pregnancy is related with the infant stool microbiome in a delivery mode-dependent manner. Microbiome. 2018;6:109.

18. Mitchell CM, Mazzoni C, Hogstrom L, Bryant A, Bergerat A, Cher A, et al. Delivery Mode Affects Stability of Early Infant Gut Microbiota. Cell Reports Medicine. 2020 Dec 22;1(9):100156.

19. Chu DM, Ma J, Prince AL, Antony KM, Seferovic MD, Aagaard KM. Maturation of the infant microbiome community structure and function across multiple body sites and in relation to mode of delivery. Nature Medicine. 2017 Mar;23(3):314–26.
