# Supplementary figures and images for: Human microbiome variation associated with race and ethnicity emerges as early as 3 months of age
Source: PLoS Biol. 2023 Aug 17;21(8):e3002230. doi: 10.1371/journal.pbio.3002230 (PMC10434942; doi:10.1371/journal.pbio.3002230)

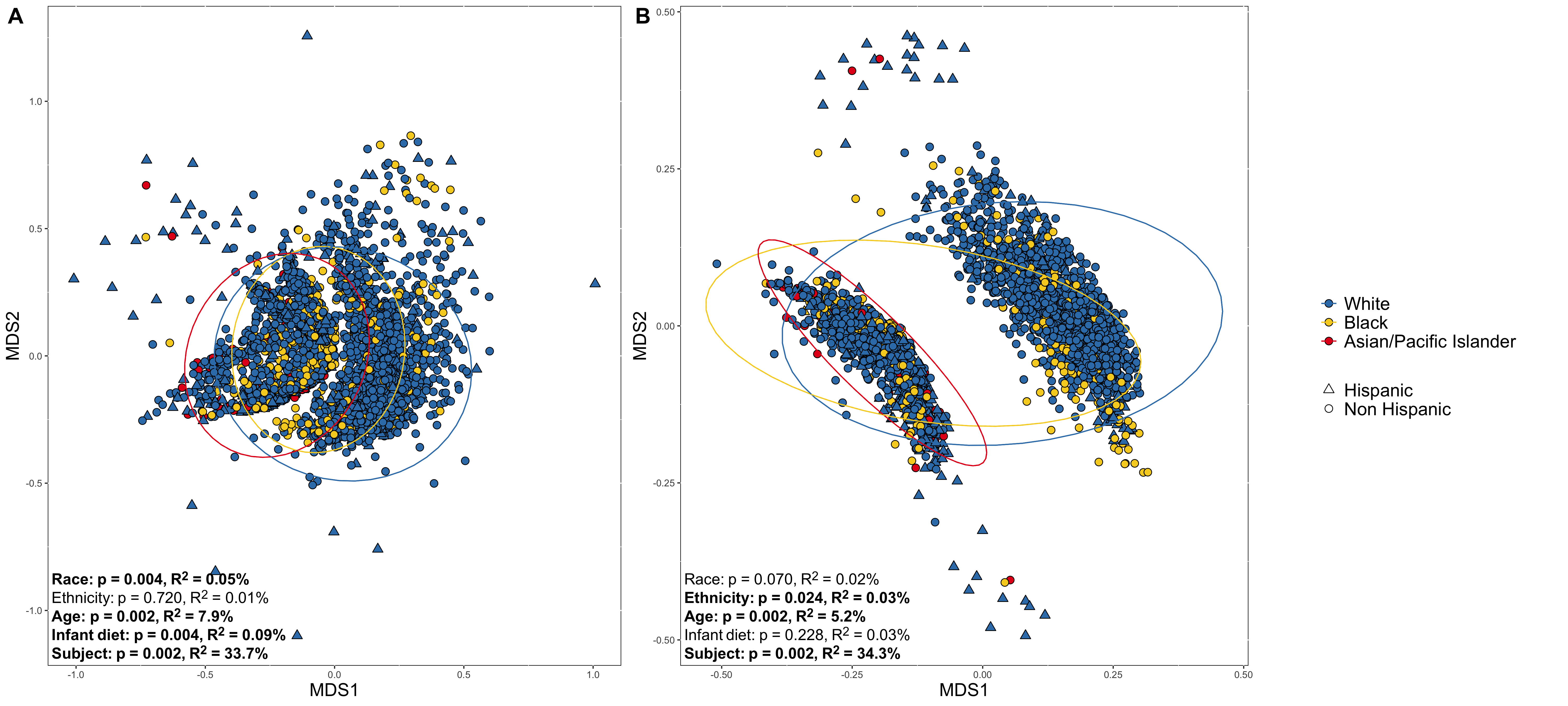

Supplement: S1 Fig — Nonmetric multidimensional scaling plots showing the effect of race on weighted (A) and unweighted (B) UniFrac distances in all samples combined. Data underlying this figure can be found in S2 and S3 Data. (TIF) [file pbio.3002230.s002.tif]

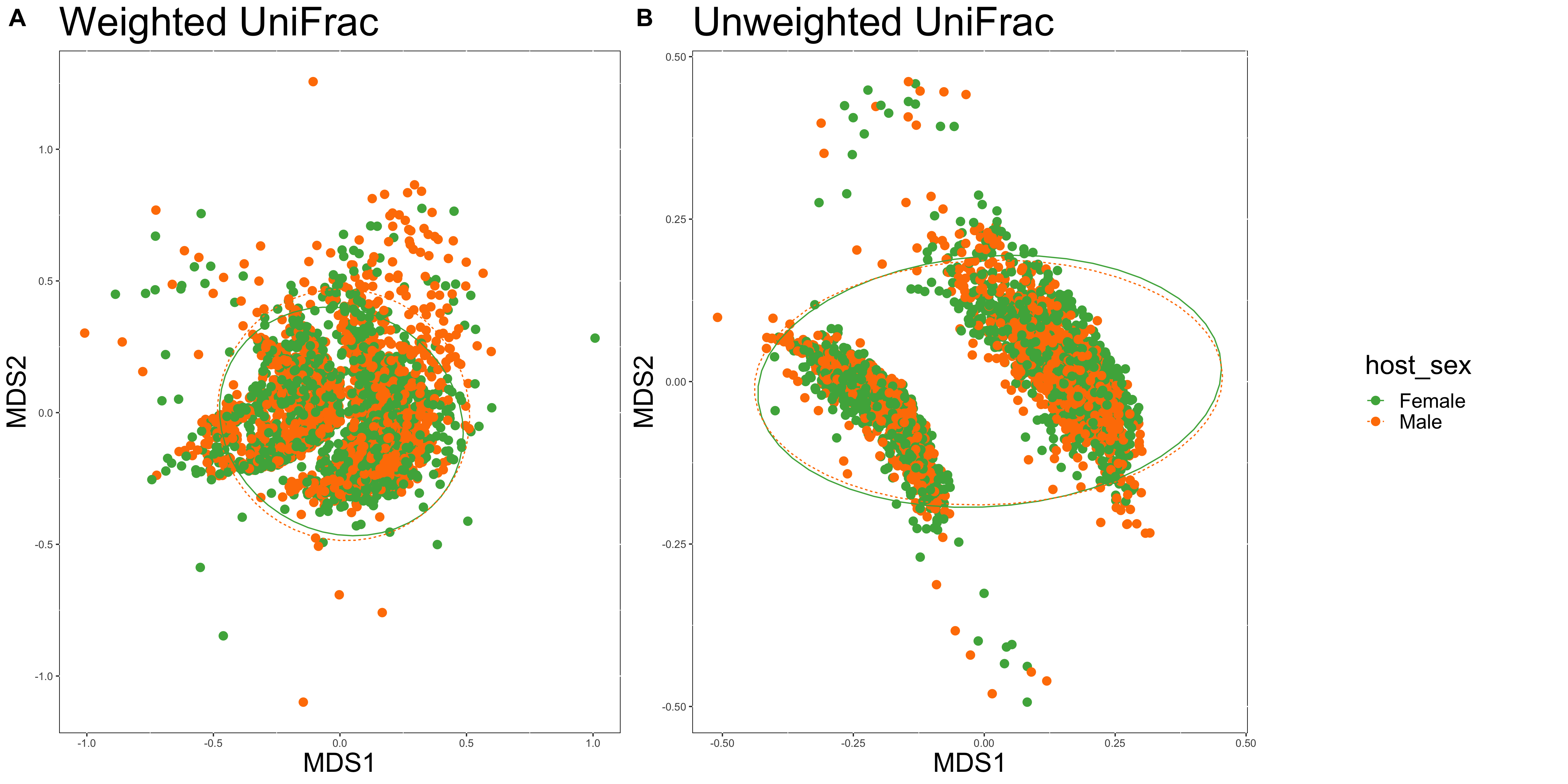

Supplement: S3 Fig — Nonmetric multidimensional scaling plots showing the effect of sex on weighted (A) and unweighted (B) UniFrac distances. Data underlying this figure can be found in S2 and S3 Data. (TIF) [file pbio.3002230.s004.tif]

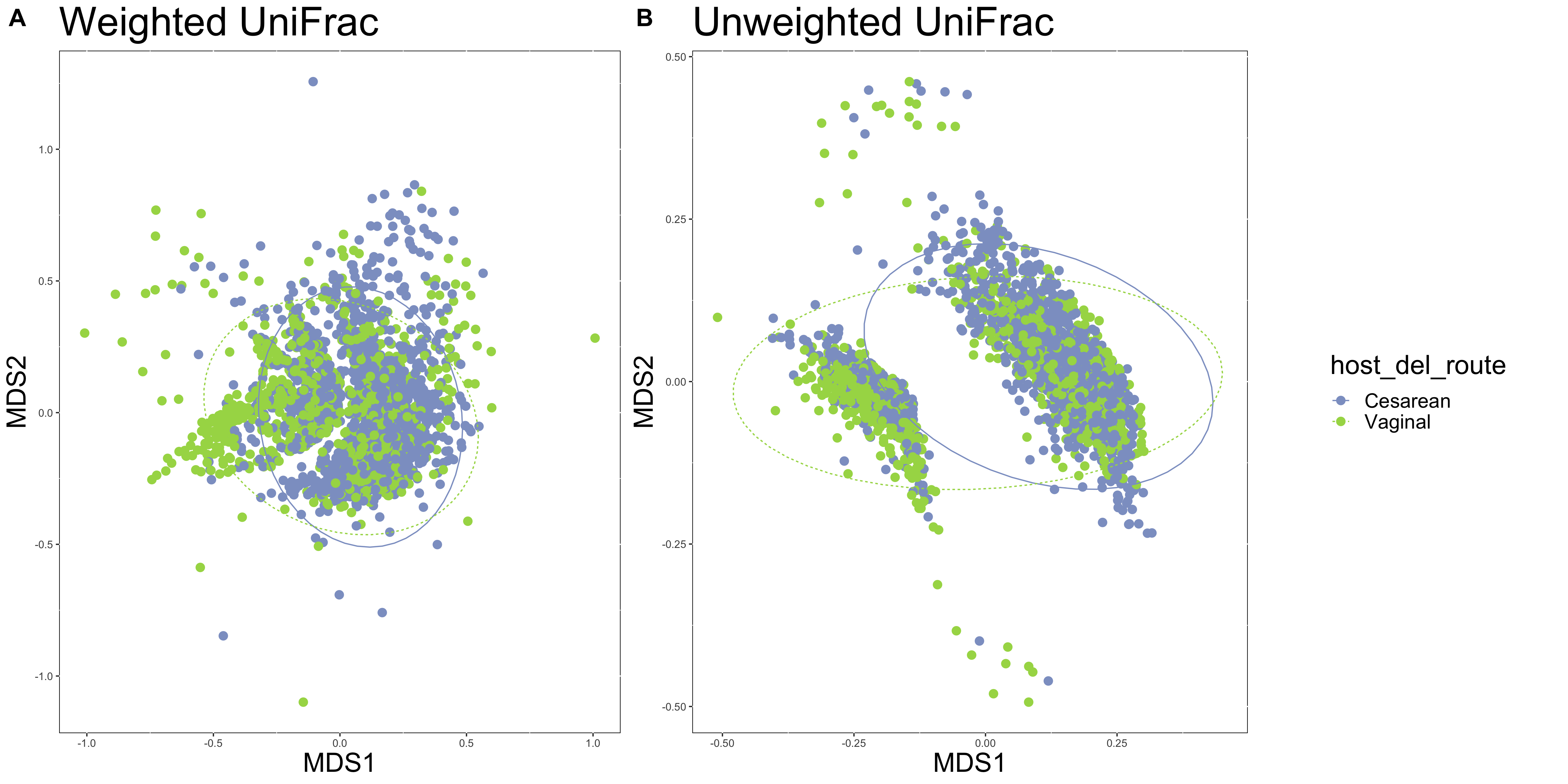

Supplement: S4 Fig — Nonmetric multidimensional scaling plots showing the effect of delivery mode on weighted (A) and unweighted (B) UniFrac distances. Data underlying this figure can be found in S2 and S3 Data. (TIF) [file pbio.3002230.s005.tif]

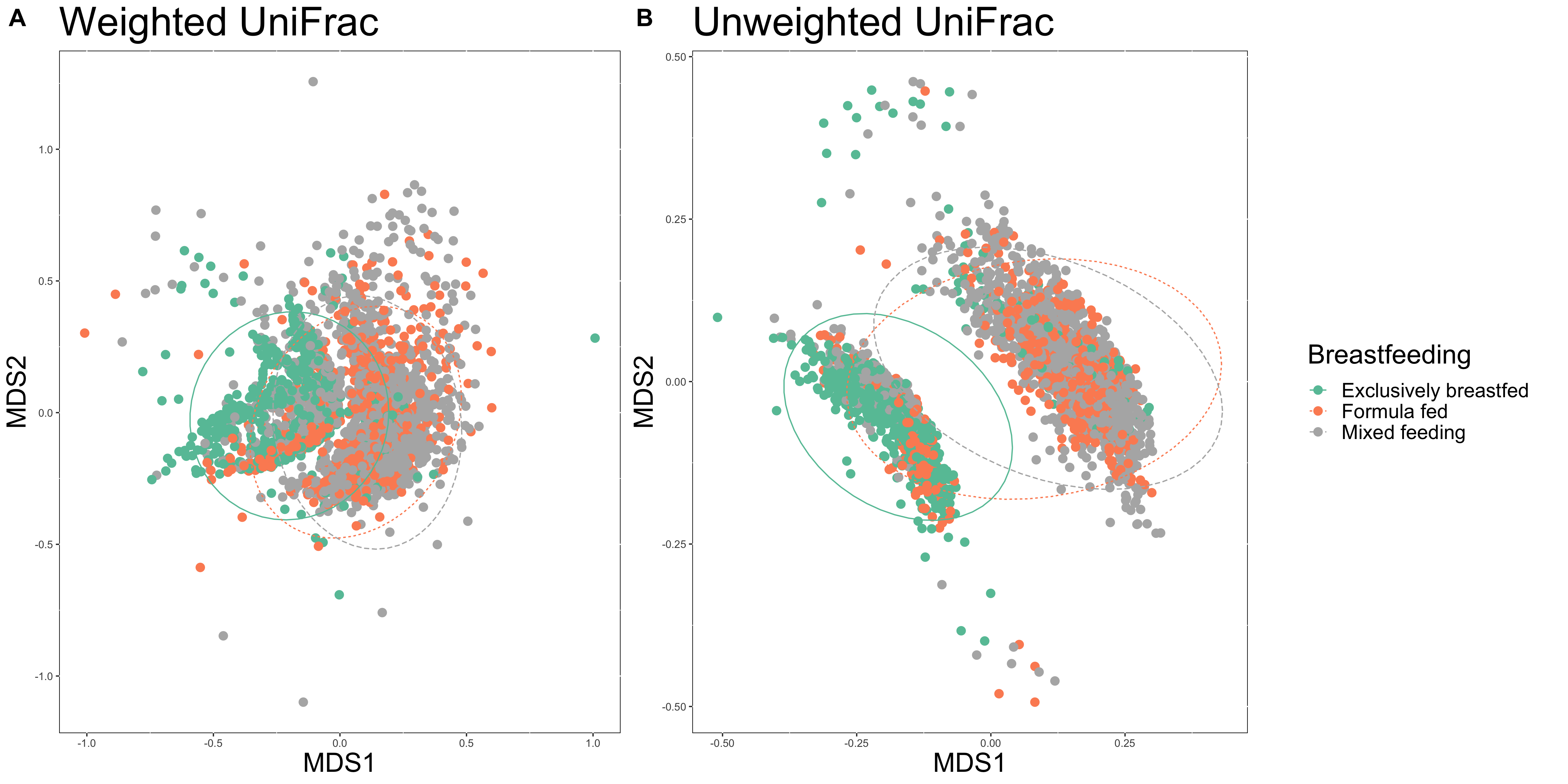

Supplement: S5 Fig — Data underlying this figure can be found in S2 and S3 Data. (TIF) [file pbio.3002230.s006.tif]

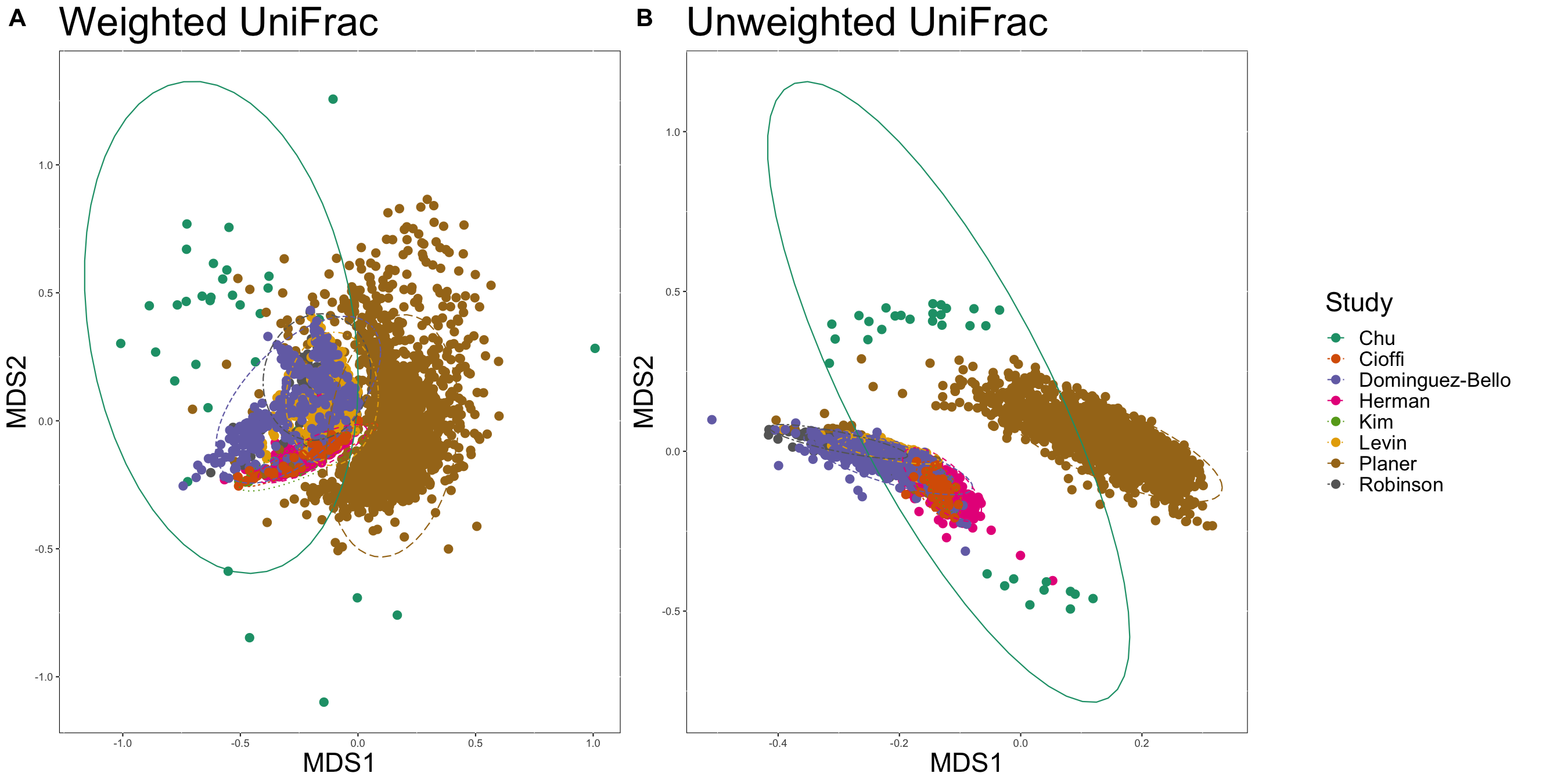

Supplement: S6 Fig — Data underlying this figure can be found in S2 and S3 Data. (TIF) [file pbio.3002230.s007.tif]

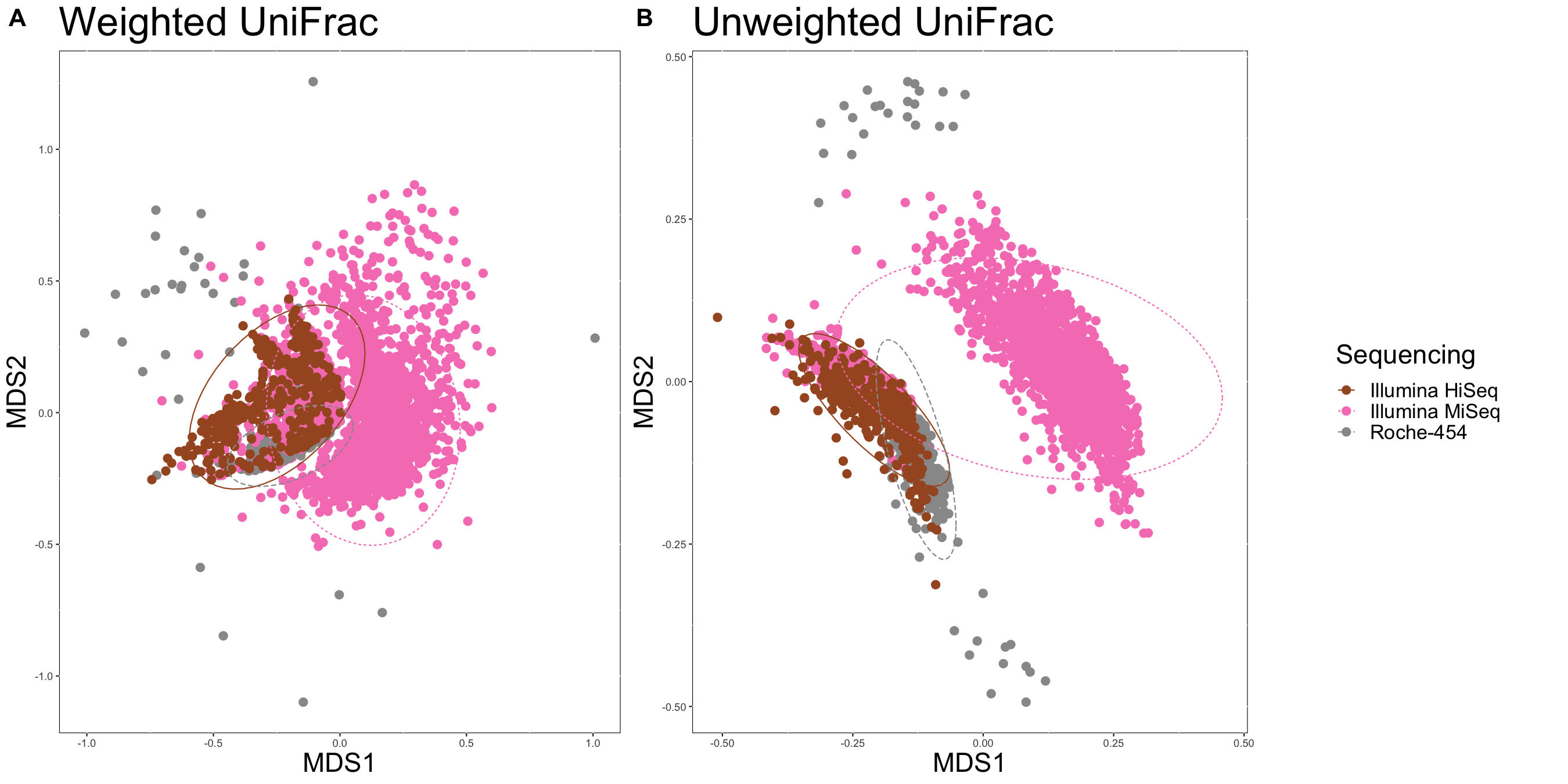

Supplement: S7 Fig — Data underlying this figure can be found in S2 and S3 Data. (TIF) [file pbio.3002230.s008.tif]

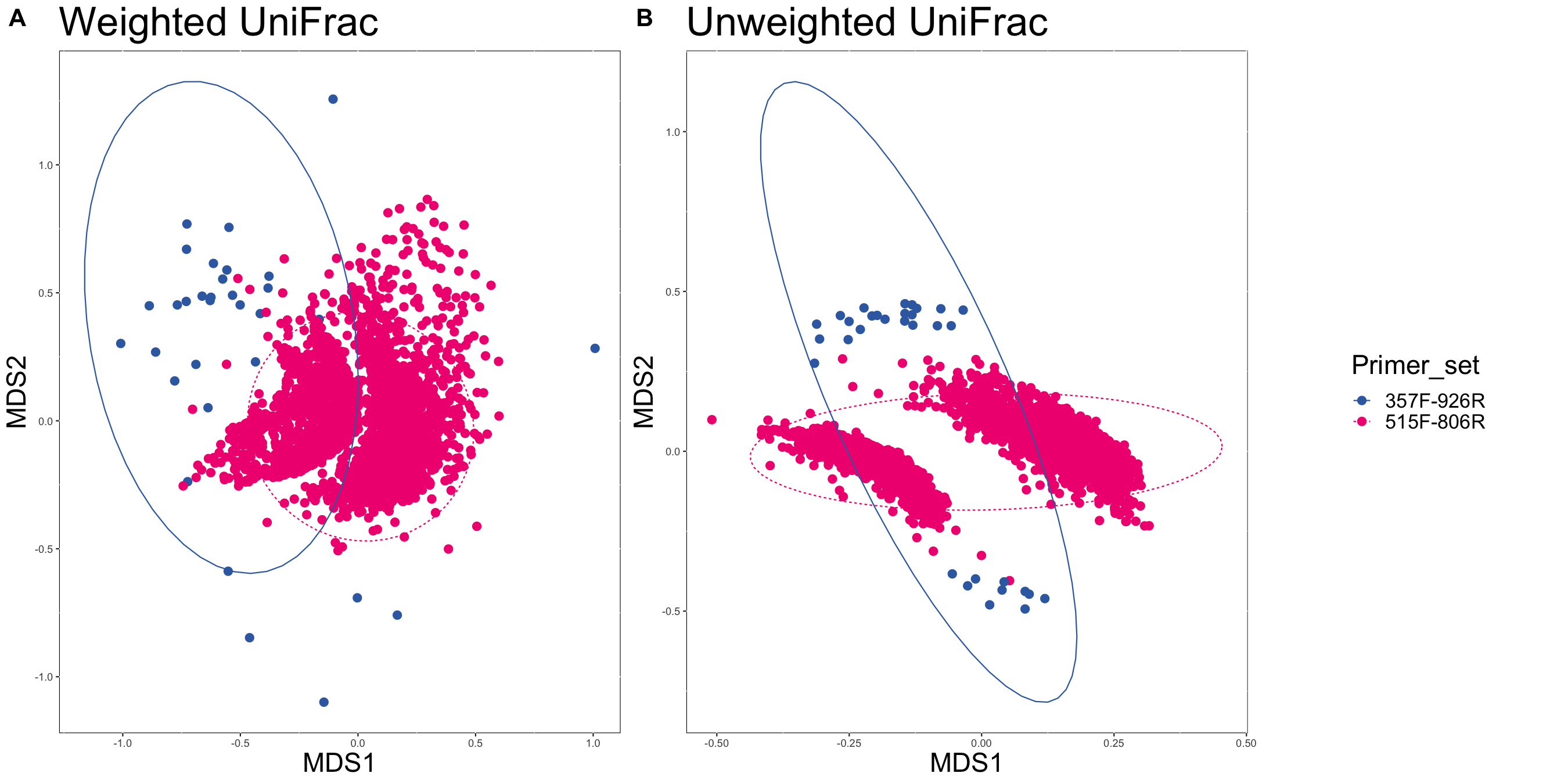

Supplement: S8 Fig — Data underlying this figure can be found in S2 and S3 Data. (TIF) [file pbio.3002230.s009.tif]

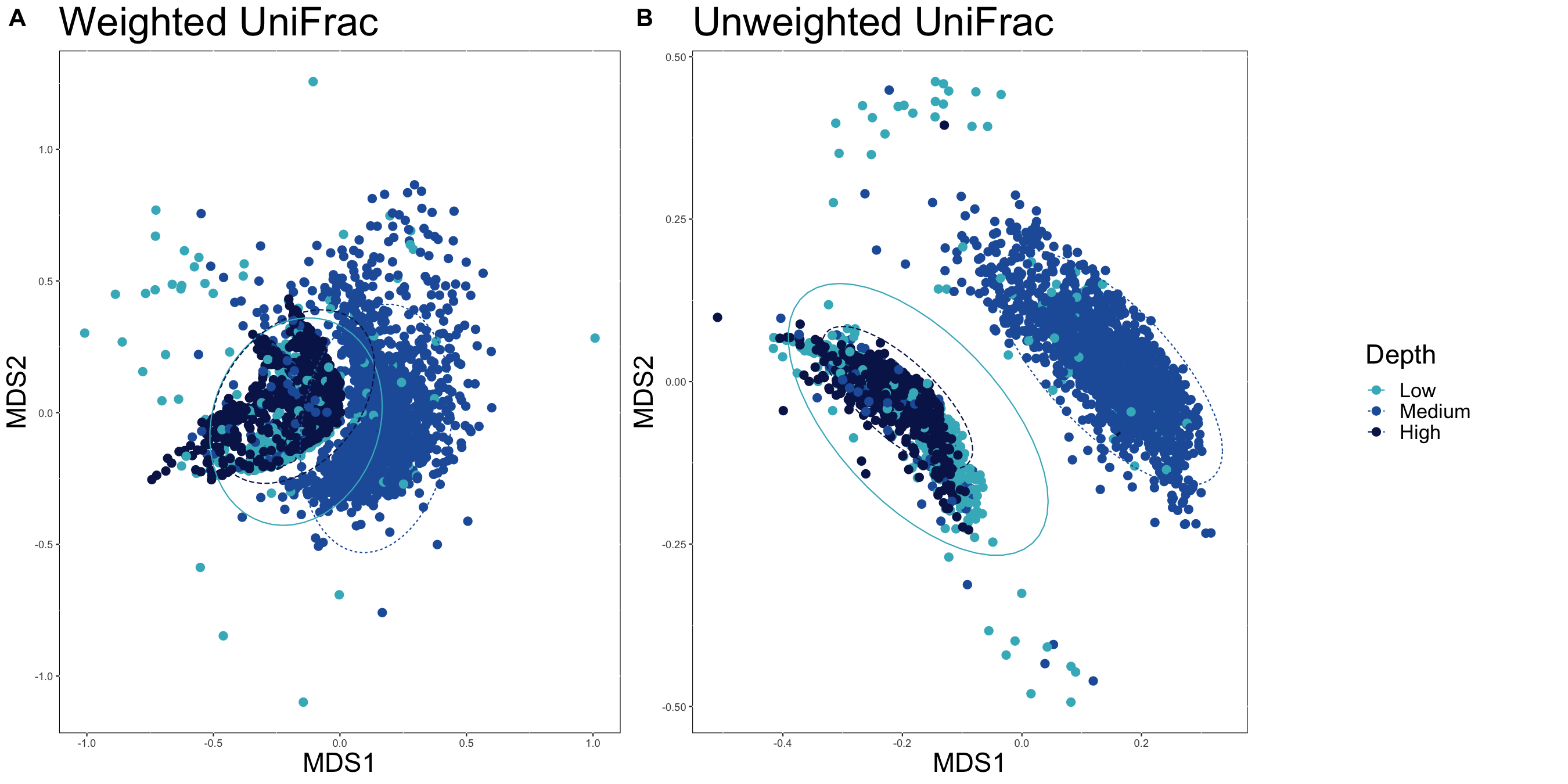

Supplement: S9 Fig — Low depth is <20,000 reads, medium depth is 20,000–49,999 reads, and high depth is ≥50,000 reads. Data underlying this figure can be found in S2 and S3 Data. (TIF) [file pbio.3002230.s010.tif]

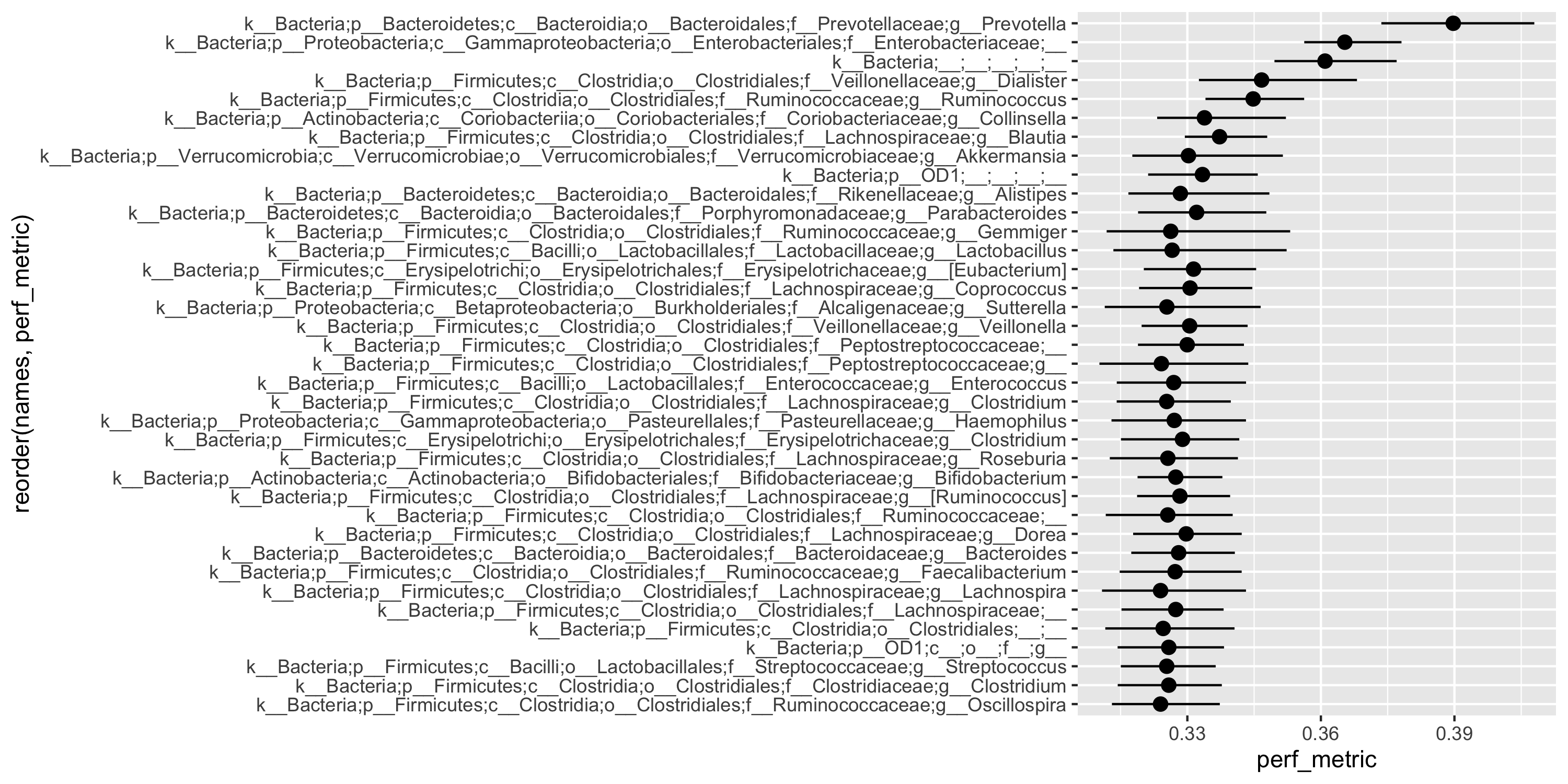

Supplement: S16 Fig — Dots denote the median importance, and whiskers denote 95% confidence intervals. Data underlying this figure can be found in S10 Data. (TIFF) [file pbio.3002230.s017.tiff]

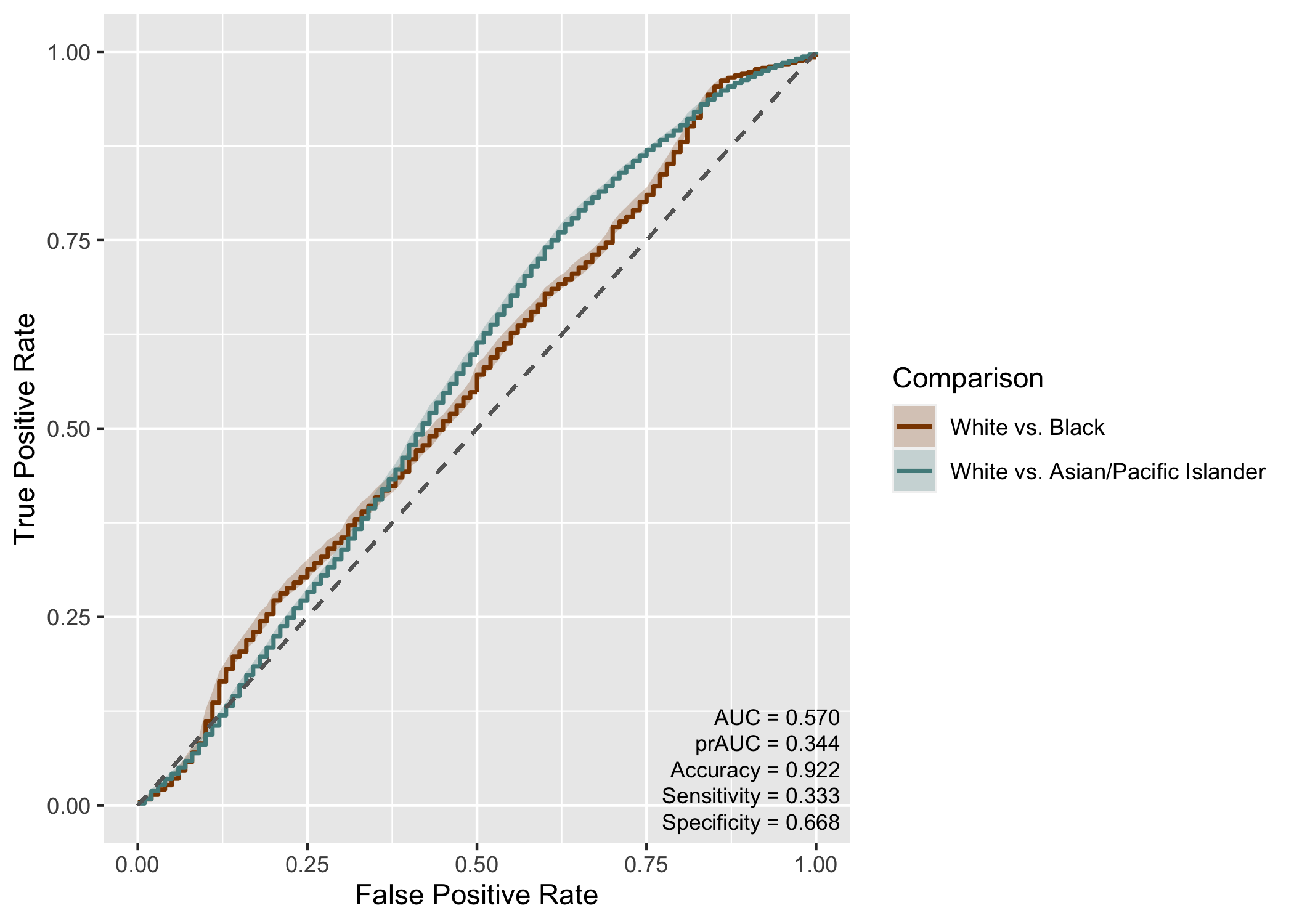

Supplement: S18 Fig — Shading represents a 50% confidence interval around the median. Data underlying this figure can be found in S12 Data. (TIFF) [file pbio.3002230.s019.tiff]

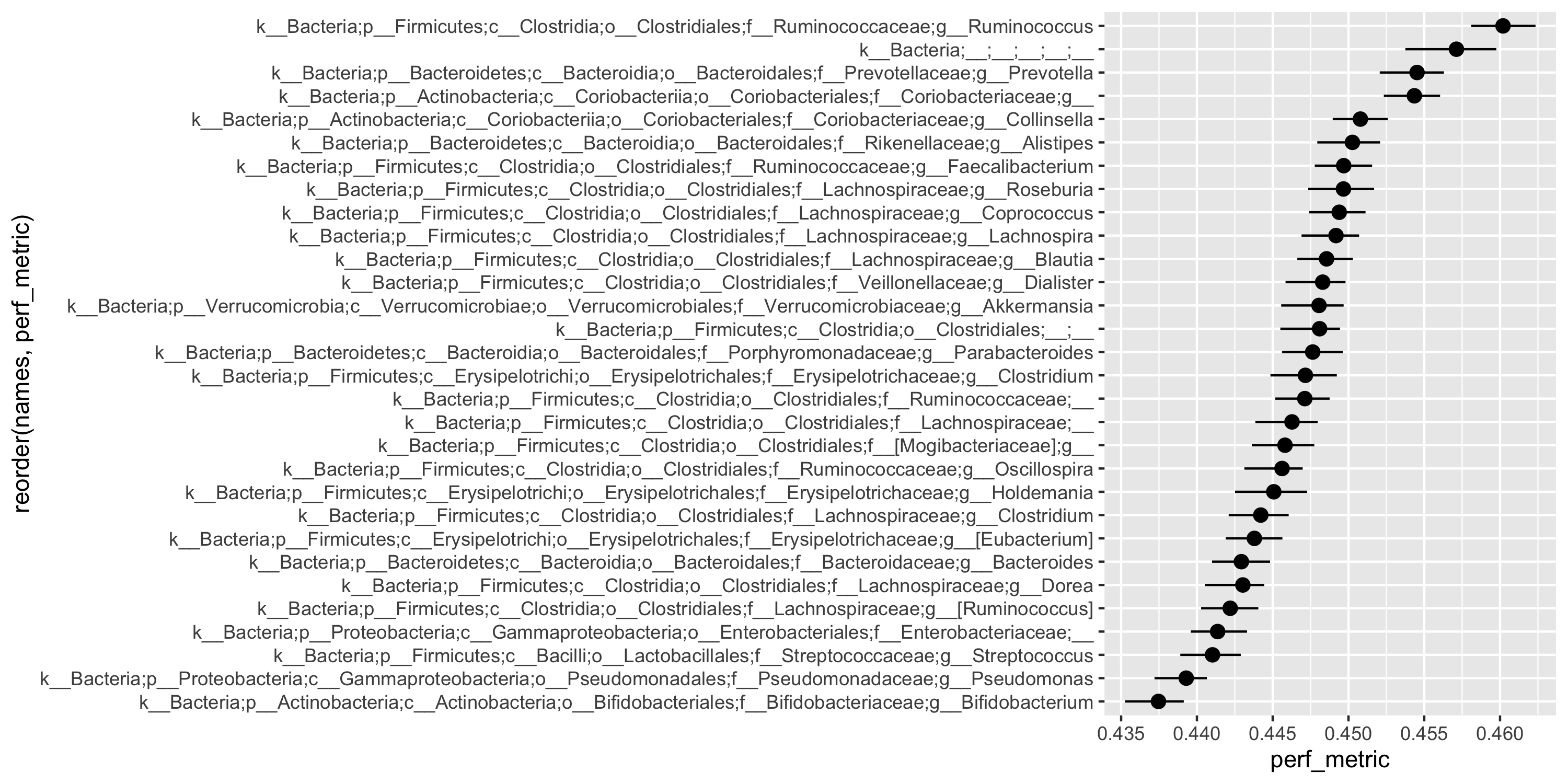

Supplement: S19 Fig — Dots denote the median importance, and whiskers denote 95% confidence intervals. Data underlying this figure can be found in S13 Data. (TIFF) [file pbio.3002230.s020.tiff]

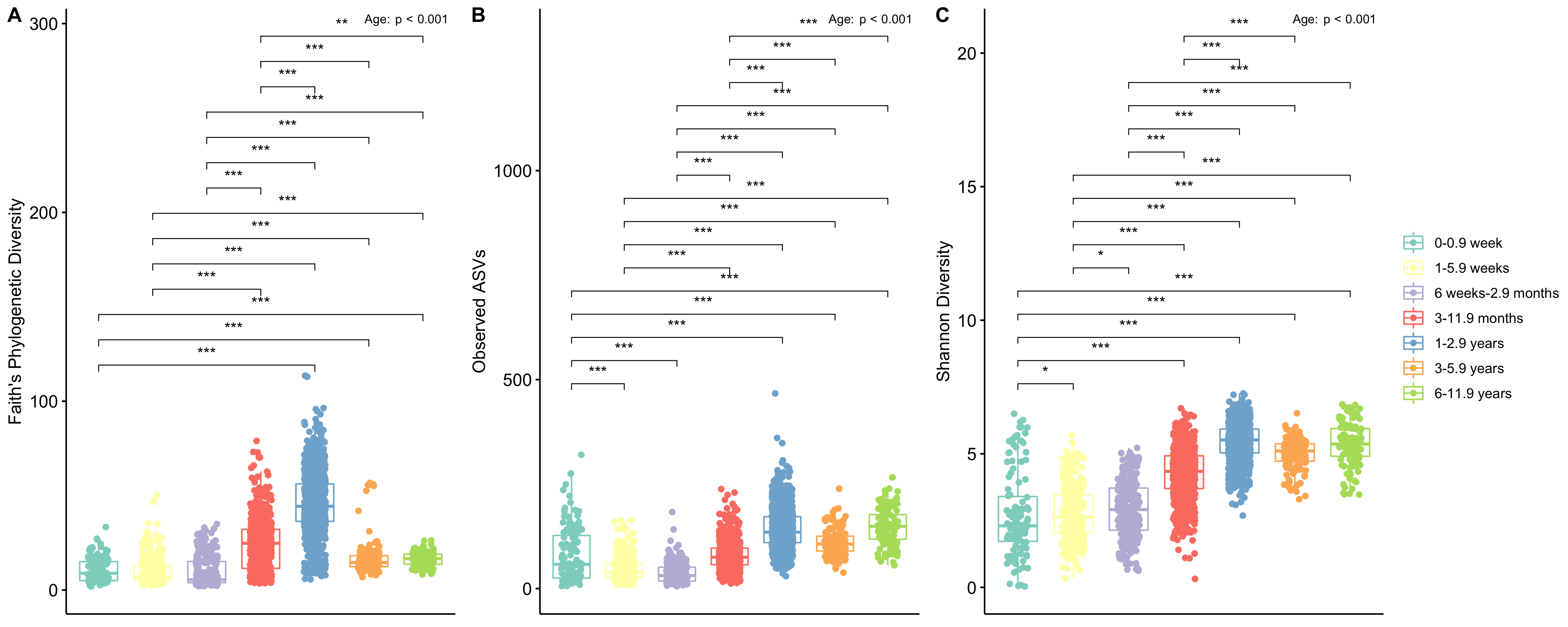

Supplement: S22 Fig — Data underlying this figure can be found in S1 Data. (TIF) [file pbio.3002230.s023.tif]

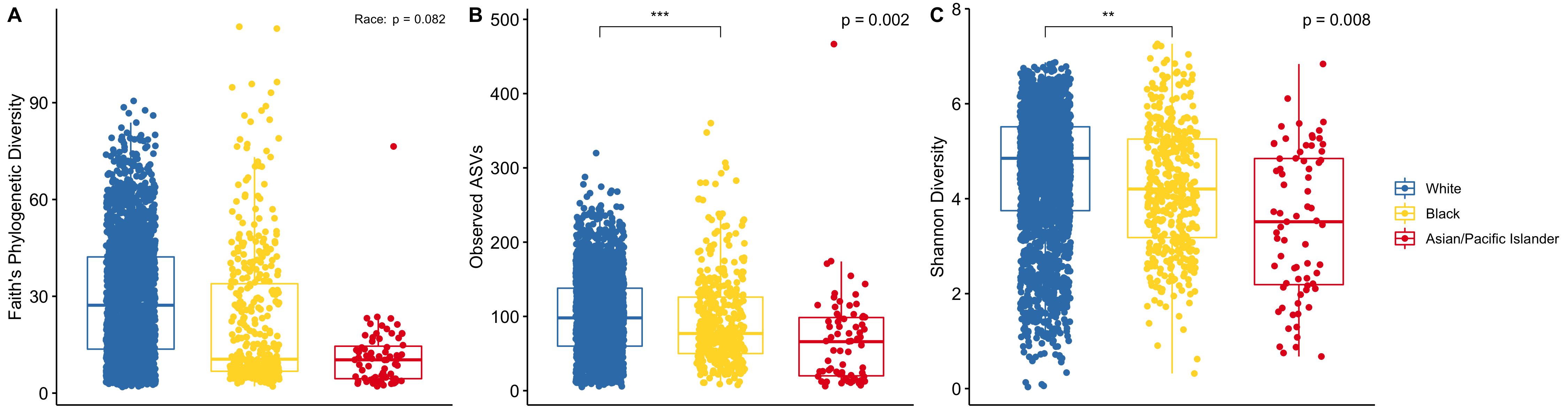

Supplement: S23 Fig — Data underlying this figure can be found in S1 Data. (TIF) [file pbio.3002230.s024.tif]

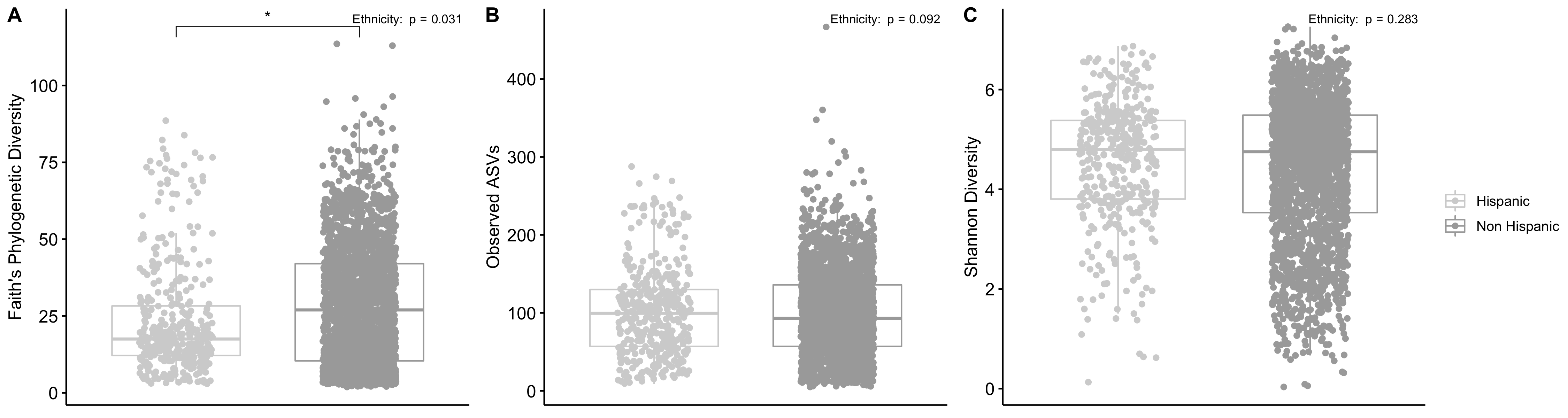

Supplement: S24 Fig — Data underlying this figure can be found in S1 Data. (TIF) [file pbio.3002230.s025.tif]

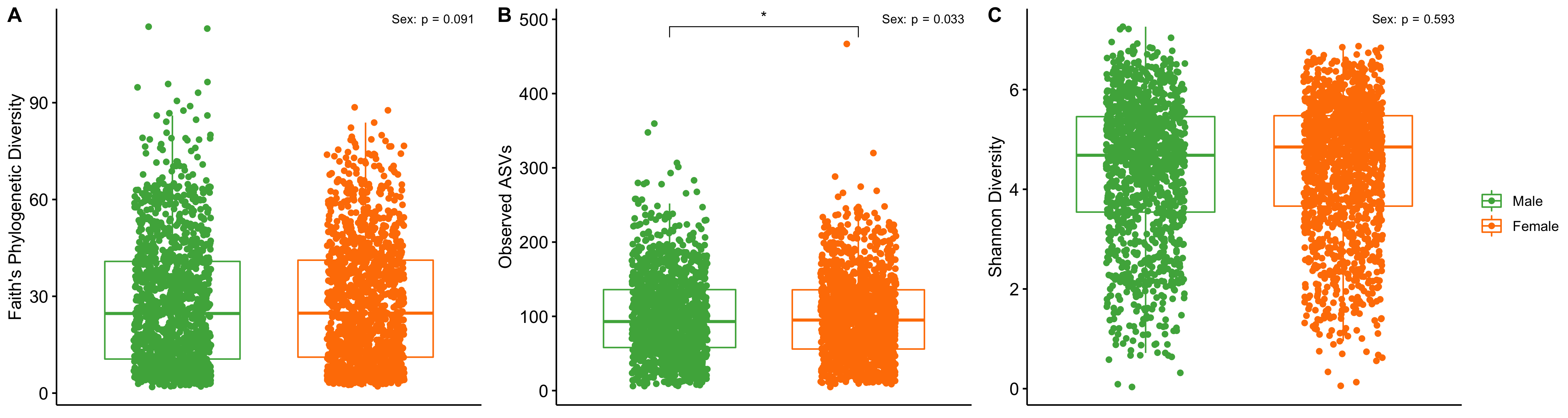

Supplement: S25 Fig — Data underlying this figure can be found in S1 Data. (TIF) [file pbio.3002230.s026.tif]

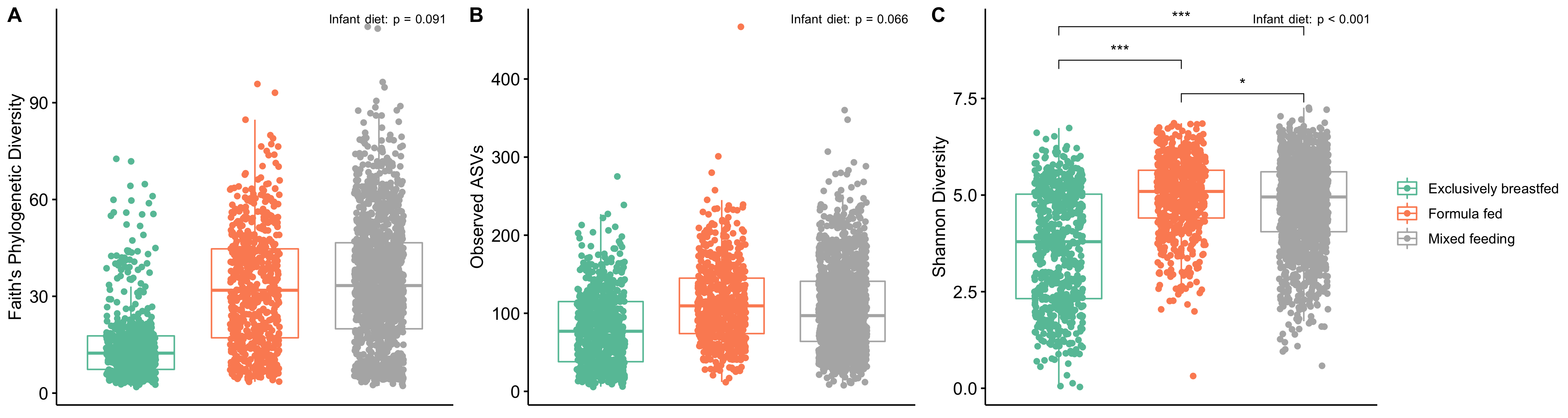

Supplement: S26 Fig — Data underlying this figure can be found in S1 Data. (TIF) [file pbio.3002230.s027.tif]

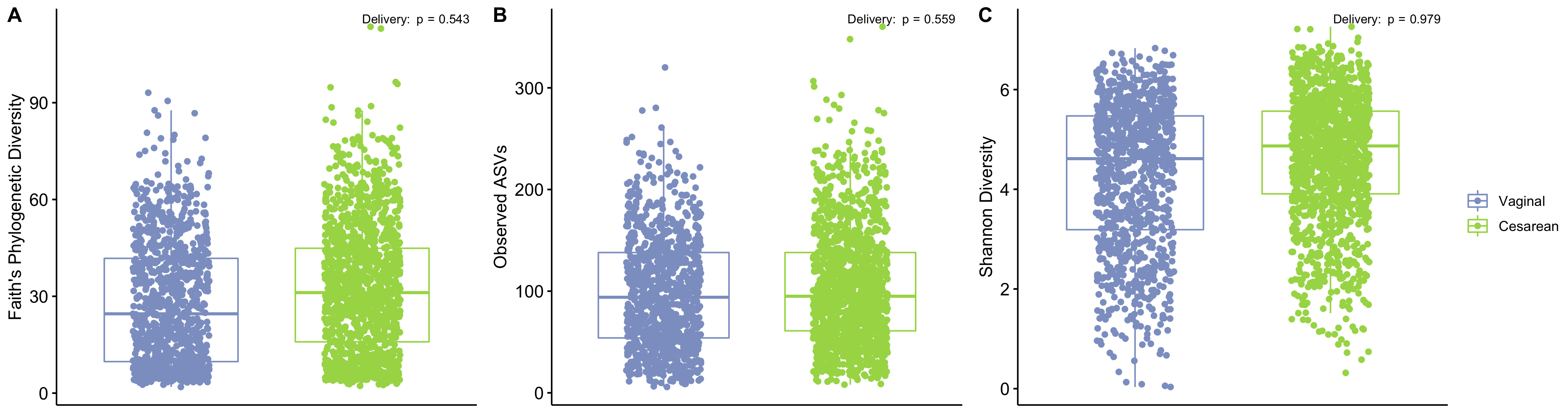

Supplement: S27 Fig — Data underlying this figure can be found in S1 Data. (TIF) [file pbio.3002230.s028.tif]
