# Supplementary material for: Human microbiome variation associated with race and ethnicity emerges as early as 3 months of age
Source: PLoS Biol. 2023 Aug 17;21(8):e3002230. doi: 10.1371/journal.pbio.3002230 (PMC10434942; doi:10.1371/journal.pbio.3002230)
Supplement: Attachment — Submitted filename: Response_to_reviewers_052223.docx [file pbio.3002230.s055.docx]

Elizabeth Mallott

Assistant Professor of Biology

[mallott@wustl.edu](mailto:emailaddress@wustl.edu)

Washington University in St. Louis

MSC 1137-0247-01

1 Brookings Dr

St. Louis, MO 63130

314.935.2058

May 24, 2023

Dear *PLoS Biology* Editor and Reviewers:

We are pleased to resubmit the following manuscript entitled **“Sociodemographic-linked microbiome variation appears after three months of age”** for consideration in *PLoS Biology*.

Thank you all for the constructive and careful feedback. We have revised our discussion of the results to address both reviewers’ concerns. In addition, we added the requested differential abundance analyses for each age group and included alpha and beta diversity analyses of study specific variables. We hope you find these updates suitable for publication. A point-by-point response to both reviewers’ comments can be found below.

The authors declare no competing interests. All authors have approved the manuscript for submission and the manuscript has not been published or submitted for publication elsewhere. Thank for considering our manuscript for publication. If you require additional information, please contact me.

Sincerely,

Elizabeth Mallott, Ph.D.	Seth R. Bordenstein

Assistant Professor	Huck Professor of Microbiome Sciences

Department of Biology	Microbiome Center

Washington University in St. Louis	Penn State University

**Reviewer #1:** The authors present a meta-analysis of existing datasets with the thesis that differences  exist in the gut microbial communities of children driven by factors captured either by race self-identified race and ethnicity or socio economic status (SES). While the authors would have preferred to use comprehensive measurements of SES, this was not possible due to lack of available metadata from the chosen manuscripts. This problem is common, and the authors instead used available data, and to their credit identify signals connecting race/ethnicity to differences in the gut microbiome. There is some literature on this that the author's appropriately reference, though this literature base needs to continue to grow.

The authors major thesis is that differences in the gut microbial communities of children with different self-identified race and ethnicity features emerges after the age of 3 months. The authors are capable of completing a comprehensive analyses of the gut microbiome, but major issues, which they can address, need to be considered prior to publication. It is conceivable that the short report format may be too restrictive for these authors and if the editors are amenable a longer format be made available to the authors.

Major Critiques:

-	A core thesis of this work is that differences in race/ethnicity interact with age. For this particular manuscript, this requires a direct assessment of the impact of Age on the gut microbiome at the taxonomic level is required. There is a superficial portion of this analysis already completed (see Figure 1A, Supplemental Table 10). Analysis of differentially abundant taxa that vary with age with plots of those taxa with age are critical. For example, do all race and ethnic groups start with similar levels of Bifidobacterium (linked to breast feeding) or Lactobacillus? Are the species the same? Are there differences at the genus level? Shannon and beta diversity are key similarities at early ages the authors describe, though specific species level similarities need to be described as well. Plots of taxa that correlate with age could be plotted in the same figure where taxa that are different at later ages are plotted against age by race/ethnicity

We included age category in the original ANCOMBC analysis that tested for the effect of race and ethnicity on taxonomic abundance, and we have now added those results to the supplementary tables (Tables S6-S9).

We have added text clarifying our analysis of all age categories combined (Lines 134-138):

“Age was included as a factor in the models, and numerous taxa were differentially abundant across age categories (Tables S6-S9). The abundances of several taxa were significantly associated with race and/or ethnicity in all samples combined (Table S5-S9), including several that varied in abundance between age categories (Figures S14-S15).”

We added the requested plots showing taxa that correlate with age by race and ethnicity (Figures S14-S15).

We have also added an analysis to identify taxa that are differentially abundant by race and ethnicity in each age category. As ANCOMBC requires at least 5 samples per group, we were unable to use it for all age categories. Instead, we used a generalized linear model with a negative binomial distribution (Tables S6-S9). Results of this analysis are reported in the main text (Lines 202-214):

“To detect differentially abundant taxa within each age category, we used generalized linear mixed models with a negative binomial distribution (ANCOM-BC requires more samples per group than we had within each age category). However, few taxa were identified as differentially abundant within each age category (Tables S6-S9). No phyla or families were differentially abundant between racial and ethnic categories within any age category, and only one genus differed between White and Asian/Pacific Islander children (Tables S6-S9). Of the six species that differed in abundance between racial categories and four species that differed in abundance between ethnic categories, none were found in more than one age group (Table S9). *Coprococcus*, one of the differentially abundant taxa within a specific age group (12-35.9 months), was more abundant in Non-Hispanic children and has been previously associated both with obesity and a high fiber diet (43). The other differentially abundant taxa within specific age groups did not have clear links to health-related outcomes in the literature. Overall, taxa with age-associated variation did not systematically vary by race or ethnicity.”

-	The authors do not account for sequencing technology of the original studies. This should be taken into account (Illumina vs other; base pair size) in addition to depth of coverage and type of sequencing (metagenomic vs 16S). These variables should be incorporated and their contributions to variations explained in Figure S6. In addition sampling depth of each study should be plotted and this data made available.

We choose to use “strata = study” to control for study-specific differences in sequencing technology, primer sets, and sequencing depth (as well as sample collection methods, sample storage, extraction methods, and specific PCR cycling conditions). More information on this approach has been added to the methods (Lines 377-385):

“Study was included in all models as strata to control for the effects of different study parameters and individual identity was included as a factor in all models to assess the impact of individual differences on microbiome communities. While sequencing method, primer choice, and sequencing depth do have a significant association with microbial community composition when included in models, including study as strata removed the effect of these study-specific parameters (Table S2). As some of the included studies had multiple participants from the same family, we also tested if individual identity or family had a larger effect size. In all cases, individual identify explained a larger proportion of the variation than family (Table S2).”

We did check that the effect of sequencing technology and primer set was no longer significant when including study as the strata but did not report those results. Those models are now included in the supplementary tables (Tables S2 and S4) and we have clarified this approach in the methods (Lines 380-383):

“While sequencing method, primer choice, and sequencing depth did have a significant association with microbial community composition when included in models, including study as strata removed the effect of these study-specific parameters (Table S2).”

We had not previously examined the effect of sequencing depth or the effect of study-specific parameters without the inclusion of “strata=study”, so thank you for that suggestion. Figure S6 and the supplementary tables have been updated accordingly. Additionally, we have added the suggested figure showing sampling depth across studies (Figure S21).

-	What is the relationship between race/ethnicity and both the variables mode of delivery and breast feeding for data where  these data are available. This is a crucial piece of information that requires visualization in this data set. For example, if breast feeding and mode of delivery are very different in one of the analyzed groups, that would be a key thing to highlight. This could reflect the biologic basis of differences between groups detected later on. This data is available in some of the tables (S11) though needs to be visually represented in a figure.

We have added figures representing this information (Figures S10 and S11), and included a reference to them in the text (Lines 333-335):

“Factors that are known to impact gut microbiome composition and were included in our models – age, sex, delivery route, and infant diet – were not independent of race and/or ethnicity (Table S5 and Figures S10 and S11).”

We also noted in the results when differences in rates of breastfeeding or mode of delivery may have impacted our findings (Lines 80-81; Lines 138-147):

“Delivery route and infant diet were not included in the age-stratified analysis, as they covaried with race and ethnicity (Table S5, Figures S10-S11).”

“Taxa positively associated with breastfeeding (*Bifidobacterium, Lactobacillus,* and *Staphylococcus*) (34,35) were significantly negatively correlated with age, as expected (Table S9 and Figures S14-S15). These taxa were differentially abundant between racial or ethnic categories, likely due to differences in rates of breastfeeding across these groups (Table S5 and Figures S10-S11). Delivery route also differed between racial and ethnic categories – vaginal delivery was more likely than expected in White, Asian/Pacific Islander, and Non-Hispanic children and less likely than expected in Black and Hispanic children (Table S5 and Figures S10-S11). However, some individual species within *Bacteroides*, which is often more abundant in vaginally-delivered children (34,35), were more enriched in Black and Hispanic children (Table S9), contrary to our expectations.”

Minor issues

-	Line 74-75 "As age had the strongest association…" describe how you came to this conclusion. It looks like Subject had the greatest explained variation (Figure S2) followed by Age. Subject would be predicted to explain the greatest variation, so the conclusions that Age was the next largest variable (and largest for the logical argument of the authors) is reasonable but the rationale needs to be more explicit. I don't disagree with the interpretation but the reader will benefit a lot from understanding the logic here.

We have clarified this in the manuscript (Lines 75-76):

“Subject explained the greatest proportion of variation, consistent with other studies of the gut microbiome (Figure S1). As age had the second strongest association with gut microbiome composition of the variables tested (Figure 1, Figure S1-S9, Tables S2-S4)….”

-	Use different symbols in Figure 2B as it is not clear. Try letters or open and closed shapes. These shapes are very difficult to discern at the size presented in the MS.

We have updated Figure 2B in the main text and Figures S12 and S13 in the supplement using triangles and circles as the shapes. We hope these are easier to distinguish. We have included the new figure legend below.

-	Table formatting is very limited. While the structure of the formatting is OK, borders, headers, shading all need to be added to make these tables easier to review. There is a lot of good information here, and it is a testament to the authors they include this material, but it needs to be cleaned up heavily for formal publication.

Tables have been reformatted to make them easier to read.

-	Strata was used for PERMANOVA analysis. This is a fair approach. The authors should list an example of the specific equation in their methods

We have added this to the methods (Lines 406-407):

“(example model: WeightedUniFrac ~ Race + Ethnicity + Age + Sex + Delivery route + Infant diet + SubjectID, strata = Study).”

-	The random forest methodology appears sound, though the method text should be clarified. What was repeated 100 times? Can the authors use the data from one study to predict the sample race/ethnicity of another study? If this cannot be done, it is only a minor issue, but if can be done it would add considerably to the manuscript.

We have clarified how we performed the random forest classification (Lines 416-419):

“Random forest classification was performed using the *mikropml* package (99) in R. 100 training/test data splits were used for each model, and 5-fold cross-validation was repeated 100 times for each of the 100 training/test data splits using the default settings of the *run_ml()* command.”

We were not able to identify an additional publicly available dataset of the gut microbiomes of children to use to test the predictive power of our random forest model, although we agree that this would have made a great addition if it were possible.

**Reviewer #2:** Mallot et al. provide a meta-analysis of 16S gut microbiome composition datasets for humans under 3 years old across racial and ethnic categories to show that differences arise shortly after birth between these groups.  Machine learning is used to show that thse differences can be used to classify the groups and the features important in these models correspond to those used to classify adult identity.  Together the data are consistent with the differences previously described in adults arising early in life.

Major comments

The presentation and discussion of the data seems biased. The authors should be careful in mis-interpretation or over-interpretation, particularly in connection to disease, since this may be counterproductive in the long run for this important field.   There is discussion and references in the taxonomic differences results section (starting at line 114) with regard to various taxa that are enriched or depleted and their associations with allergy, etc consistent with the authors' premise.  However, earlier in the manuscript the authors show that Black children have higher alpha diversity, yet the abundant literature that associates alpha diversity with health and low diversity (seen in the white children here) with many inflammatory diseases is not discussed or referenced.  Therefore, the data appears to be sending a mixed signal that the authors gloss over.

Thank you for this critique, as we did not intend to bias the presentation and discussion of the data. We have modified our discussion of the results to take greater care when discussing possible links to health conditions. Our aim was to highlight that we are finding potentially health-relevant differences, but that we cannot explicitly link our childhood results to adult health outcomes and that our findings are mixed – we are finding both negative and positive characteristics of the microbiome in all race and ethnicity categories.

For the alpha diversity results, we have tried to better contextualize the results by adding the following text (Lines 93-97):

“While higher alpha diversity is consistently associated with better cardiometabolic health and lower incidence of inflammatory disease in adults (28–30), studies have found mixed results in children. For example, studies of associations between alpha diversity and risk of allergic disease have found negative (31), positive (32), and no (33) association.”

When discussing taxa and their relationship to allergic and autoimmune disease, we have added the following text to better contextualize the results (Lines 194-197):

“Thus, we find higher relative abundances of at least one taxon that is positively associated with health in Asian/Pacific Islander, Black, and White children, highlighting the complexity of linking the relative abundance of individual gut microbial taxa to health as a whole.”

Another example is in lines 161-4 and figure S14:

"In contrast, Ruminococcus is specifically important in the child-adult models, likely due to similar variation in abundance between racial categories in both children and adults (Figure S14). Higher abundances of Ruminococcus are linked with an increased risk of colorectal cancer (37), a disease for which there is a known racial health disparity (38).

One would expect based on this statement that Ruminococcus is more abundant in Black and Asian/Pacific Islander, however in S14 it appears to be more abundant in white for both children and adults. If this taxon's contribution is due to enrichment in whites, why do the authors point out its association with diseases that are associated with health disparities.  The data appears to support the opposite, so please clarify.

When discussing the *Ruminococcus* results, we edited the text to highlight the fact that our finding was the opposite of what one might predict (Lines 260-268):

“Higher abundances of *Ruminococcus* are linked with an increased risk of colorectal cancer (46), a disease for which there is a known racial health disparity (47,48); however, we find that *Ruminococcus* is most abundant in White individuals, a group whose colorectal cancer risk is lower than that of Black individuals but higher than that of Asian/Pacific Islander individuals. Race-associated variation in the relative abundance of *Ruminococcus* across adult guts is not universal, is likely due to a subset of *Ruminococcus* species, and may interact with other factors such as stress or BMI (1,49). Thus, it is difficult to know how or if the differences observed in the microbiome here contribute directly to health disparities.”

Another important point is that the authors appear to neglect the major taxonomic shift that is known to occur in infants upon the introduction of solid food.  The final part of the paper that examines differences in taxa is performed across all ages.   Since the infant microbiome from 0-3 years can be thought of as existing in two states, with some transition between them -- pre-solid food and post-solid food.  While the timing of solid food introduction may not be available, the authors may use other criteria to create these two bins, such as their initial analysis, changes in abundance of key taxa known to change developmentally, or an age that would generally make sense as a cut-off.  You could imagine 0-6 months as pre-solid food, 6-12 months as a transition (maybe don't use this data) and >12 months as post-solid food.

Breaking down this analysis is important for multiple reasons since the initial beta-diversity analysis indicates that the significant differences appear at later ages.  This separation of early and late may also enable detection of early life taxonomic differences that currently go undetected due to the larger effect and noise introduced from the data of older children.

We are happy to include additional age breakdowns. This dataset spans a period when the recommended age for the introduction of solid food varied from 4-6 months, so we’ve included 0-3.9 months (definitely pre-solid food) and 4-11.9 months (transition). 1-2.9 years was already included as an age category for alpha and beta diversity analyses. As also requested by reviewer 1, we conducted the taxonomic differences analysis for all age groupings. Additional categories for the alpha and beta diversity analysis can be found in Tables S2 and S4, and the age-specific differential abundance analyses can be found in Tables S6-S9.

Specific points

If publications exist where these datasets were first reported, please include in table S1. Also include the author identifiers (eg "Kim") in the columns so this can help track the datasets between figures and the table.

This information has been added to Table S1.

The main message of the supplemental figs S1-S6 appears to be that study appears to correspond to the separation of the two clusters in weighted and unweighted (S6).  Since there is an uneven distribution of data across studies, particularly at the extreme young and old ages (table S1), the authors should explain how this was accounted for in the analysis to avoid study-specific artifacts.

We used “strata = study” in the PERMANOVAs to control for study-specific differences in the beta diversity analyses and included study as a random effect in the alpha diversity analyses. More information on this approach can be found in our response to Reviewer 1.

Why are the colors different between the weighted and unweighted in S6?  For example, there is a lot of brown in the left graph and a lot of green in the right graph.  Are these not the same data from the studies plotted in two different ways?

It seems we had some differences in the colors assigned to the individual studies in that graph (likely due to not specifying the order of levels for the Study factor). We have corrected this.

L 85. "Pairwise comparisons

86 confirmed that Black individuals had higher within-sample diversity than White individuals at 3-

87 11.9 months and 1-2.9 years".

Would be good to add "for at least one of the five measures of diversity" to recognize this doesn't reflect all measures.

We have added the following update (Lines 91-92):

“Pairwise comparisons confirmed that Black individuals had higher within-sample diversity than White individuals at 3-11.9 and 12-35.9 months for at least one of the five measures of diversity (Figure 2A, Table S4) (27).”

Fig. S7-S8.

-Why are there more age bins created for the unweighted (S8) vs. the weighted (S7)?

The other groupings for weighted UniFrac are included in Figure 2 in the main manuscript.

-The groupings in the weighted appear to match the study specific effects.  For example, the collapse of the two clusters to one main cluster in the last two groups may correspond to the oldest group having data that is dominated by one study. So are the two groupings in the younger plots (2.9 years and younger) reflective of study-specific differences or race and ethnicity?  Perhaps there is a better way to present the data?

You are correct that the two clumps correspond to study-specific differences (which are statistically accounted for by using strata in the PERMANOVAs). We struggled with how to best present the unweighted UniFrac data; we did not want to hide the clear effect of study, but also know that it is dominating the plots. We could not come up with an alternative (other than solely presenting the weighted UniFrac data). We are quite receptive to additional reviewer suggestions on how best to address this.

Fig. 2 legend should be reorganized so the text associated with each panel is separated.  Currently the information for A and B is intertwined and it is a bit confusing.

The Figure 2 legend now reads:

“**Figure 2.** **Microbiome variation emerges at or shortly after 3 months of age.** (A) Dot and whisker plots show estimates for Tukey pairwise comparisons in the alpha diversity linear mixed effects models. Dots indicate the estimated difference in alpha diversity when accounting for other covariates in the model, whiskers denote 95% confidence intervals, and the dashed line indicates zero or no difference. Comparisons with whiskers that do not cross zero indicate a significant difference in alpha diversity between those two categories. Colors in the dot whisker plots denote alpha diversity metric, and dot shape and line type denote age category. (B) NMDS plots show weighted UniFrac distances between by race and ethnicity at 0-2.9 months, 3-11.9 months, and 12-35.9 months. Colors and 95% confidence ellipses in the NMDS plots denote race, and shape denotes ethnicity. Blue text in the panels highlights significant p-values. NMDS plots for additional age categories and unweighted UniFrac distances can be found in the supplementary material (Fig S12-S13).”

"0-2.9 months of age, 3-11.9 months of age, and 1-2.9 years of age".  The discontinuity of numbers and units is a bit awkward.  Recommend keeping everything in months and making the last category 12-35.9 throughout the figures and text.

This has been modified both in Figures 1 & 2 and throughout the text.

To identify differentially abundant taxa by race and ethnicity, the authors perform an analysis across all age categories.  This is perhaps not so intuitive for the reader considering the previous section showing that younger ages do not have significant differences.  To avoid skepticism of post-hoc decisions, the authors should either clearly articulate rationale for why they included the younger ages or present supplemental data showing how exclusion of the younger ages impacts the results.

We have added differential abundance analyses within each age category (see response to Reviewer 1).

L 121. "Four of the 19 overlapping taxa were higher in abundance in both Black children and

122 adults compared with White children and adults, and four of the overlapping taxa were lower in

123 abundance in both Black children and adults."

What about the 11 overlapping taxa that are not mentioned.  If 8 show similar enrichment patterns, but 11 show opposite patterns, what does this say about the concordance between analyses/studies?  Please clarify how this is different from random.

Thank you for the opportunity to clarify. We did not mean to suggest that the pattern is or is not different from random. Our goal was to highlight taxa that overlap and have a similar effect in both studies. We have provided additional information to describe the directionality of the relationships for the other 11 taxa to clarify (Lines 169-173):

“The remaining 11 overlapping taxa were either differentially abundant between either Asian/Pacific Islander children and Black children or Asian/Pacific Islander children and White children, or the direction of effect differed between Black and White adults and children. Among the eight taxa that overlapped and had the same effect in children and adults…”

L 126. "these taxa have

127 been associated with an increased risk of autoimmune and allergic diseases, asthma, and obesity

128 across human populations (28-32)."  Should be "across industrialized human populations". This is important to note since so many non-industrialized populations are enriched in taxa such as Prevotella copri and have low incidence of  autoimmune and allergic disease.

We agree that we should specify that we are primarily talking about people from populations that are described in much of the literature as industrialized. We have adjusted the text to read (Lines 176-178):

“These taxa have been associated with an increased risk of autoimmune and allergic diseases, asthma, and obesity across humans in Europe and North America (28,36–39).”

L 131. "Conversely, Veillonella, which decreases the risk of asthma and allergic

132 disease(28,33),"  Careful with wording since as stated, a causal relationship is implied, yet the references appear to only test associations.  Maybe, "which is associated with decreased risk of".

Thank you for noting our inappropriate use of causal language. The text now reads (Lines 181-182):

“Conversely, *Veillonella*, which is associated with a decreased risk of asthma and allergic disease (33,36), is consistently lower in abundance in White children”

L 157. "However, the taxa with the highest importance differed with

158 respect to the magnitude and direction of the differences between adults and children". I think this means that the enrichment of taxon X can help predict race Y in children, yet depletion of taxon X can predict race Y in adults.  If this is the case, it brings up the question of what this means biologically.  It would be good for the authors to discuss cases, and provide some specific examples, where taxa are important in both cases, but show different directions in adults and children.

Yes, that is what we meant. We have clarified that and added a few examples of other taxa that are important for distinguishing the gut microbiome of different groups, but show different directions in adults and children (Lines 247-256):

“Specifically, Enterobacteriaceae and *Prevotella* are highly important in child-child models but are of modest importance in child-adult models (Figures S16 and S19), and their relative abundances are lowest in White children but highest in White adults (Figure S20). Other studies have similarly found that specific taxa can be used to differentiate the gut microbiome of groups of people, but that the direction of effect can differ between adults and children. *Prevotella* was highly important in both adult and child random forest models used to detect taxa that distinguish the gut microbiome across geographic regions, but the direction of the differences in relative abundance differed (44). In children, *Prevotella* was more abundant in the United States, but *Prevotella* was more abundant in adults outside of the US (44). *Alistipes* was found to be protective against IBS in adults, but predictive of IBS in children (45).”
